# Outcomes of left atrial appendage closure versus oral anticoagulant therapy in patients with atrial fibrillation: an updated meta-analysis of randomized control trials

**DOI:** 10.1186/s43044-024-00576-1

**Published:** 2024-10-22

**Authors:** Ketut Angga Aditya Putra Pramana, Ni Gusti Ayu Made Sintya Dwi Cahyani, Yusra Pintaningrum, Basuki Rahmat

**Affiliations:** 1 General Practitioner, Faculty of Medicine Mataram University, Mataram, Indonesia; 2 Interventional Cardiology Division, Cardiology and Vascular Department, Faculty of Medicine, Mataram University, Mataram, Indonesia

**Keywords:** Atrial fibrillation, Left atrial appendage closure, Oral anticoagulant

## Abstract

**Background:**

The purpose of this study is to compare the clinical results of Left Atrial Appendage Closure (LAAC) and oral anticoagulation (OAC) in individuals with AF.

**Methods:**

For randomized controlled trials (RCTs) comparing the clinical results of OAC to LAAC in patients with atrial fibrillation (AF), we searched PubMed, ScienceDirect, and Cochrane. The included publications were subjected to meta-analyses using Review Manager v5.4.

**Results:**

In comparison to OAC, LAAC was linked with a decreased incidence of all stroke (OR 0.68; 95% CI 0.55–0.84; *p* = 0.0004). LAAC was also linked to a decreased risk of hemorrhagic stroke (OR 0.20, 95% CI 0.07–0.55; *p* = 0.002). There is no statistically significant difference between the two groups in terms of ischemic stroke (OR 1.05; 95% CI 0.59–1.84; *p* = 0.88) or systemic embolization (OR 1.02; 95% CI 0.42–2.46; *p* = 0.97).

**Conclusions:**

According to our meta-analysis, the LAAC was less likely than the OAC to have a complete or hemorrhagic stroke. For the two groups, however, there was no difference in the risk of ischemic stroke or systemic embolization.

## Background

About quarter of ischemic strokes are caused by atrial fibrillation (AF), a common atrial arrhythmia brought on by the left atrial appendage (LAA). Embolic strokes are common in these cases [1–3]. OAC has been demonstrated to be efficacious in avoiding ischemic stroke in AF patients [4]. Inadequate management of the international normalized ratio in patients on vitamin K antagonist therapy, drug discontinuation, underdosing, and noncompliance with prescription guidelines, on the other hand, are hurdles to OAC [5].

Recent research has demonstrated the efficacy of OAC and left atrial appendage closure (LAAC) devices in avoiding stroke in patients with atrial fibrillation (AF) [6]. Despite the fact that OAC has been shown to lower the risk of strokes, many patients are not excellent candidates for OAC therapy. LAAC devices were created as an alternate course of therapy as a result of these inadequacies [7]. We did this meta-analysis since significant studies evaluating the effectiveness of LAAC closure devices with pharmacological treatment had just been published [8–10]. Comparing the clinical results of LAAC with OAC in AF patients is the purpose of this meta-analysis.

## Methods

**Fig. 1:**
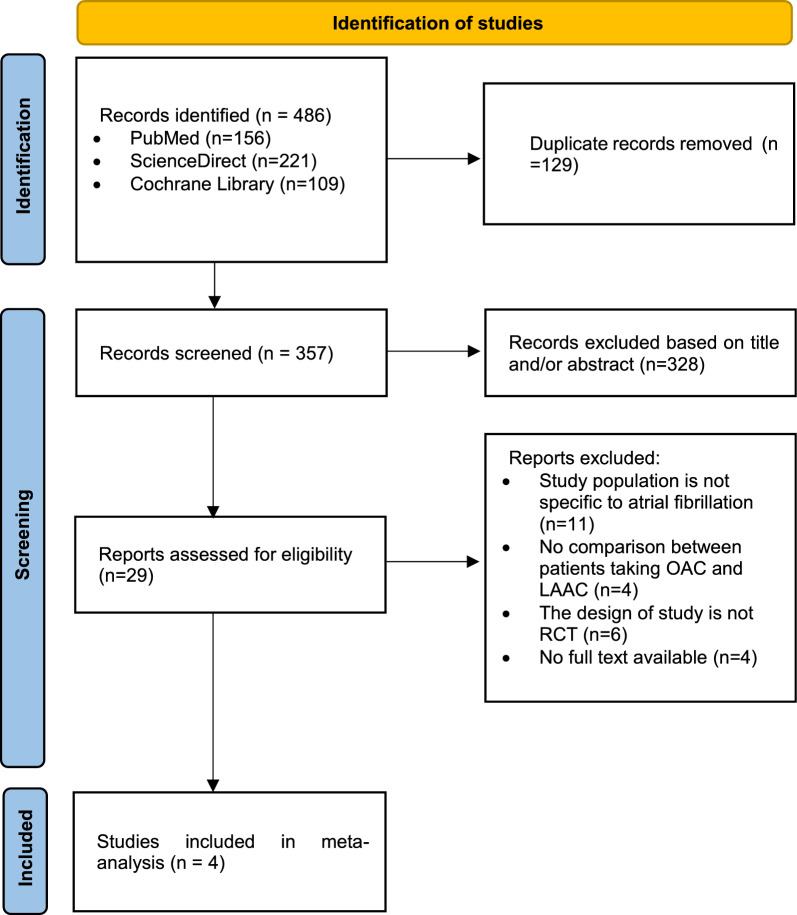
PRISMA flow chart

The current review and analysis refer to statements in the instruction of the Preferred Reporting Items for Systematic Review and Meta-Analysis (PRISMA) (Fig. 1). A detailed protocol has been registered earlier in PROSPERO CRD42023476482.

### Data sources and search strategy

We reviewed the whole body of literature in order to incorporate all pertinent research up to January 2023. We looked through the Cochrane, ScienceDirect, and PubMed databases. Studies that satisfied the following requirements were included [1]: The study examined the effects of treating AF with LAAC versus OAC [2]; it was a randomized control trial (RCT); and [3] it was published in a peer-reviewed publication. A search strategy was created for each database using MeSH phrases and Boolean operators. The terms "atrial fibrillation AND medical therapy OR oral anticoagulation OR OAC AND left atrial appendage closure OR LAAC" were included in the search.

### Study selection process

Following the elimination of duplicate entries, the initial search records were evaluated independently by KAAPP and NGAMSDC using the title and abstract as criteria. A comprehensive text analysis was then carried out. The subsequent criteria for research inclusion were employed: The research was conducted as a randomized control trial (RCT), evaluated the effectiveness of LAAC and OAC therapy for AF, and was published in a peer-reviewed publication. All parties involved, including the other author (YP), were satisfied with the resolution of disagreements.

### Outcome measures

The outcome is all strokes, ischemic stroke, hemorrhagic stroke, and systemic embolization.

### Data extraction and quality assessment

KAAPP, NGAMSDC, and BR were responsible for retrieving the baseline parameters of all included research. KAAPP, NGAMSDC, and BR then carried out a thorough quality evaluation of the included research using the risk of bias assessment for RCT studies developed by the Cochrane Collaboration. Any differences of opinion were settled by consensus, taking into account the opinions of the other author (YP).

### Statistical analysis

Odds ratio (OR) and 95% confidence interval (CI) were calculated using the Mantel-Haenszel random effects models. To measure statistical heterogeneity between groups, the Higgins *I*^2^ statistic was utilized. An *I*^2^ value of 0 suggested a lack of heterogeneity, but an *I*^2^ value more than 50% denoted significant heterogeneity. Because of the study's unpredictability, we used random-effect models to calculate the odds ratio (OR). In this work, we employed Egger's test funnel plot to estimate the likelihood of publication bias. Review Manager 5.4.1 was used for all analyses. To define statistical significance, a two-sided *p* value of less than 0.05 was employed.

## Results

### Study search and selection results

The initial search strategy turned up 486 studies. Following a comprehensive text, inspection of 29 potentially relevant papers and then four randomized control trial studies were included in this systematic review and meta-analysis (Fig. 1).

### Baseline characteristics and quality assessment

Table 1 displays the baseline characteristics of the included studies which consist of four RCT studies. Each study was a randomized controlled trial that satisfied the requirements to be included in the systematic review and meta-analysis. The four studies had 6286 participants in total; 3312 of them received a LAAC and 2974 an OAC.

**Table 1:** Baseline characteristics of included studies

References	Country	Design	Sample Size		Follow up (years)	Outcome
			LAAC	OAC		
Prague 17 [8]	Czech Republic	RCT	201	201	1.7	All strokes, ischemic stroke, systemic embolization
Prevail [12]	US	RCT	269	138	1.5	All strokes, ischemic stroke, hemorrhagic stroke, systemic embolization
Protect AF [13]	US and Europe	RCT	463	244	1.5	All strokes, ischemic stroke, hemorrhagic stroke, systemic embolization
whitlock et al*.* [14]	Canada	RCT	2379	2391	3.8	All strokes, ischemic stroke, systemic embolization

**Fig. 2:**
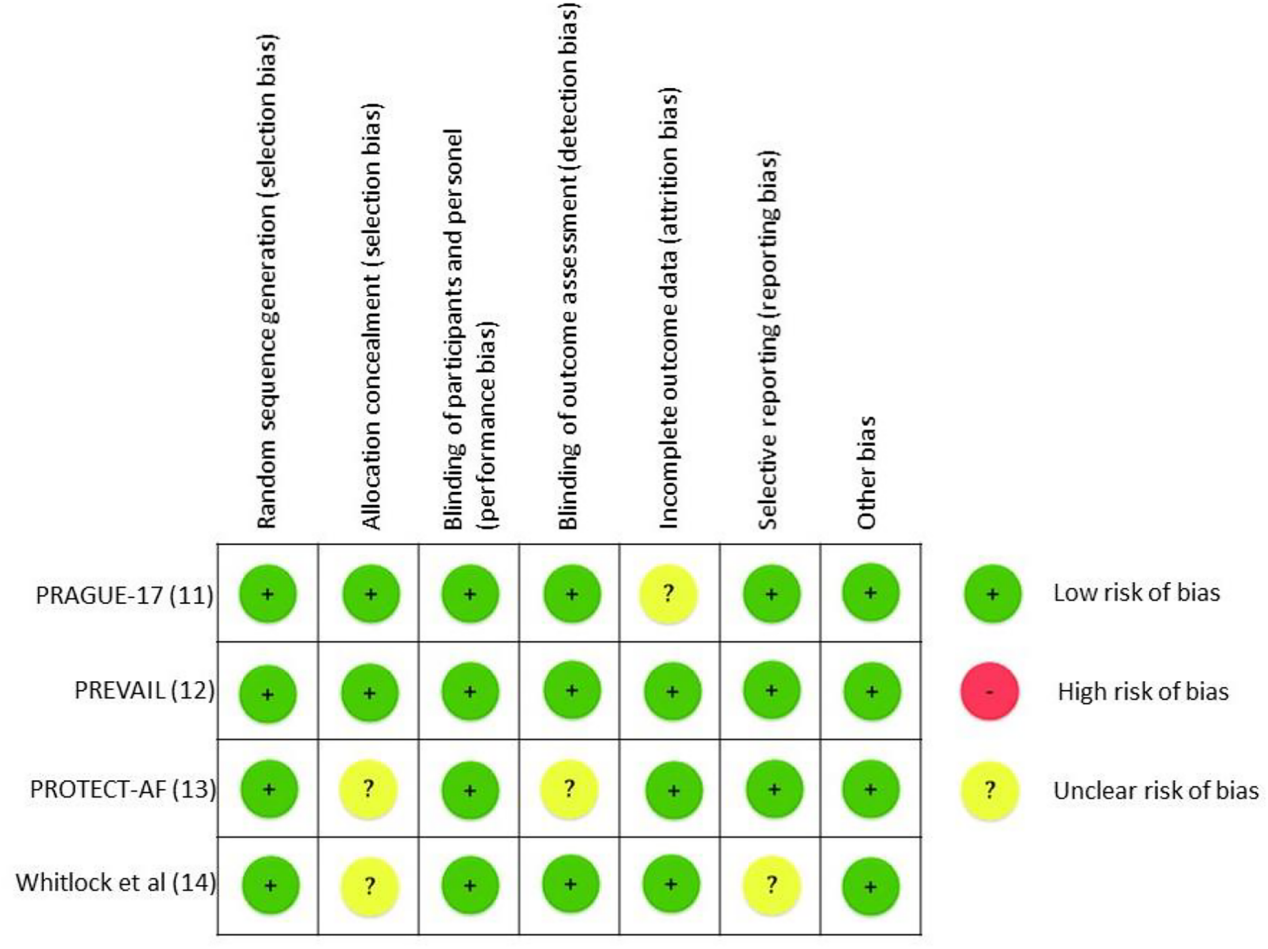
Risk of bias assessment for RCT studies

**Fig. 3:**
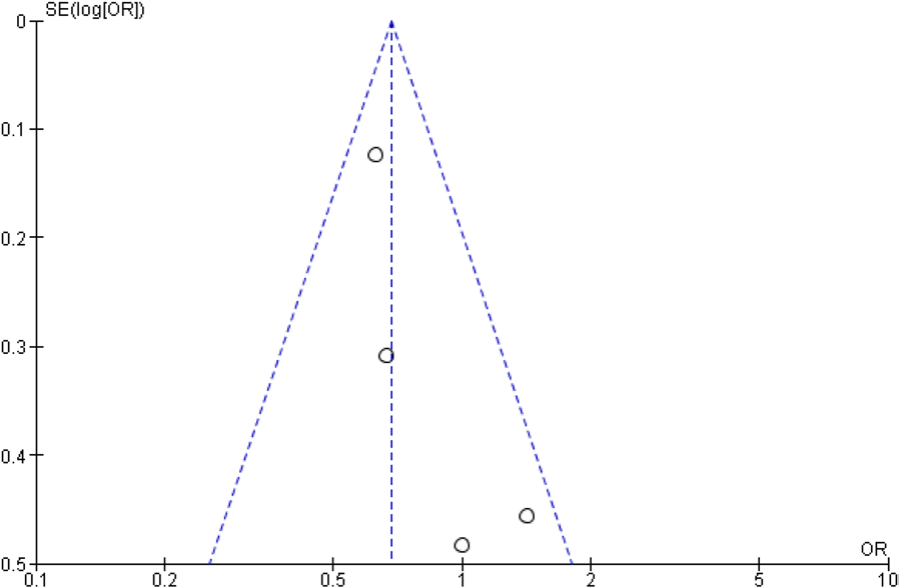
The funnel plot of the all strokes

**Fig. 4:**
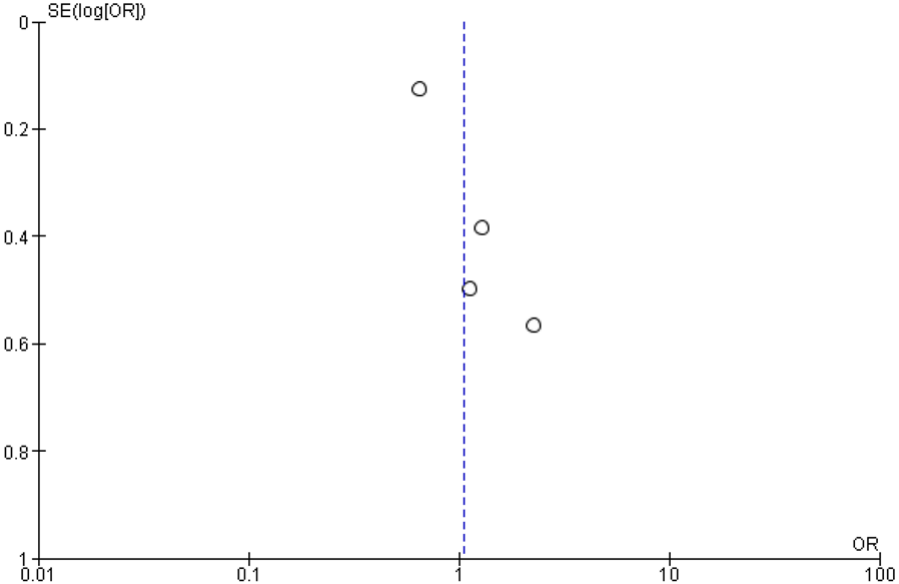
The funnel plot of the ischemic strokes

**Fig. 5:**
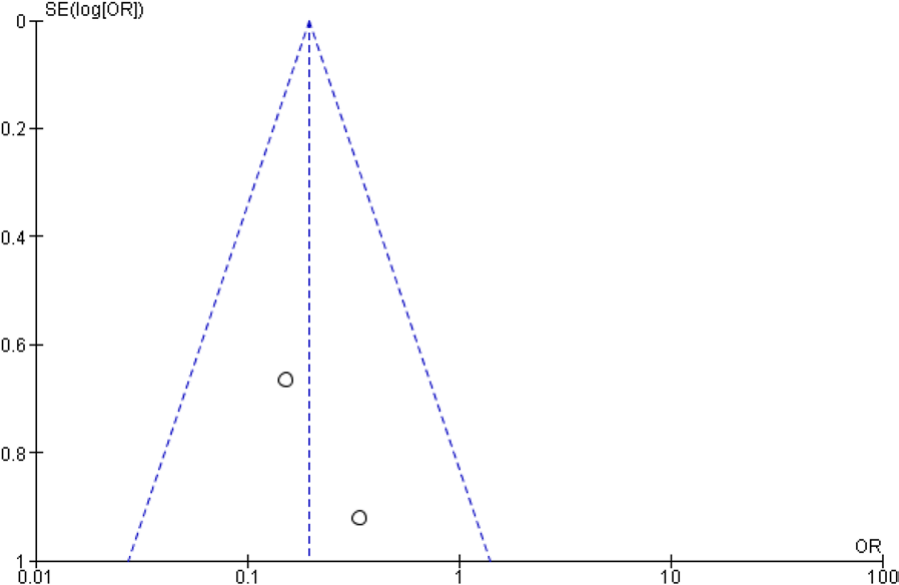
The funnel plot of the hemorrhagic strokes

**Fig. 6:**
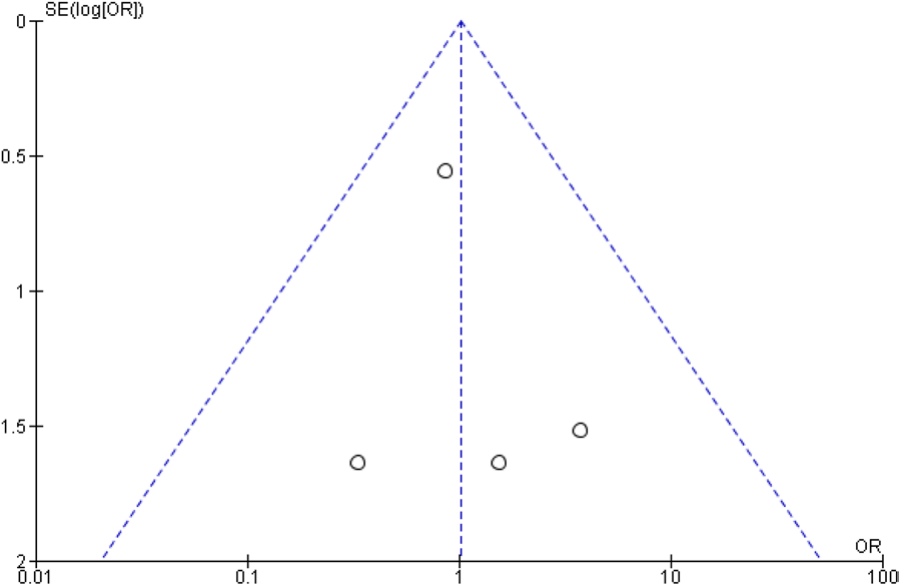
The funnel plot of the systemic embolization

The quality and bias risk were evaluated in four of the included publications using the Cochrane Collaboration's risk of bias assessment for RCT trials (Fig. 2). No RCT study has a material risk of bias. It is evident from the funnel plots in Figs. 3, 4, 5, and 6 that it is impossible to overlook the publication bias of the included papers.

### Pooled analyses for clinical outcomes

#### All strokes

**Fig. 7:**
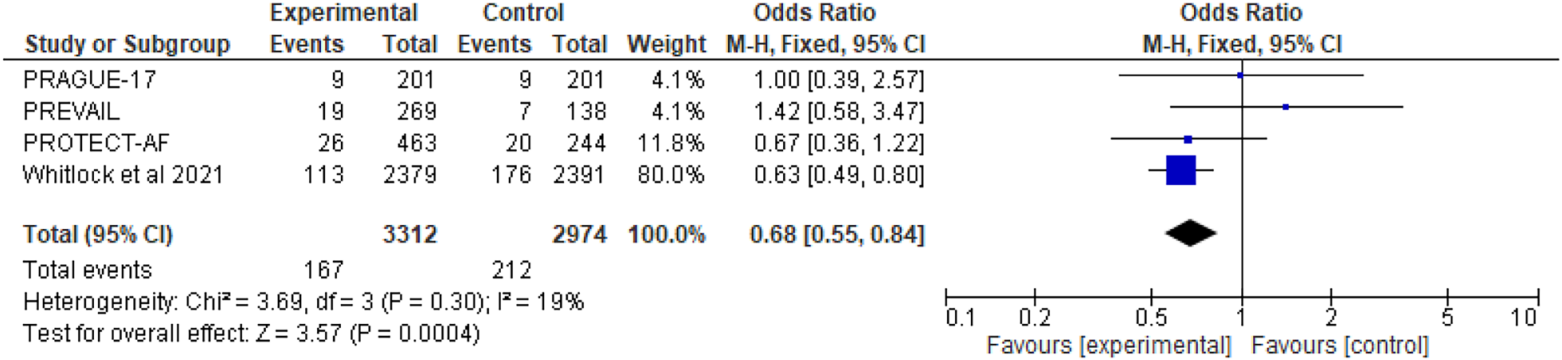
The forest plot of the all strokes

There are four RCT studies mentioned about risk of all strokes, involving 6286 patients were identified, 3312 patients received LAAC while 2974 received OAC therapy. A fixed effect model's results (Fig. 7) showed that LAAC was substantially linked to a decreased risk of all strokes when compared to OAC (OR 0.68; 95% CI 0.55-0.84; *p* = 0.0004; *I*^2^=19%).

#### Ischemic strokes

**Fig. 8:**
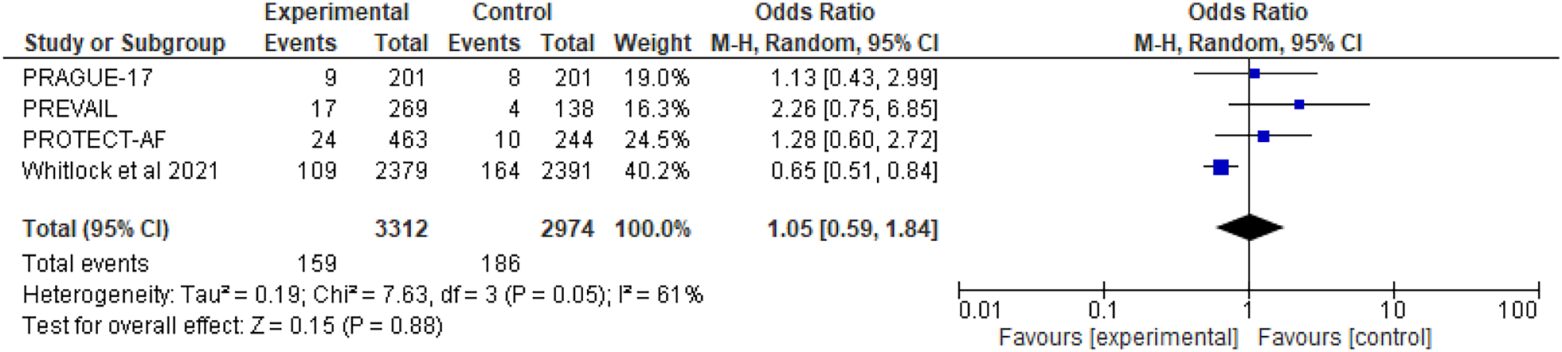
The forest plot of the ischemic strokes

There are four RCT studies mentioned about risk of ischemic strokes, involving 6286 patients were identified, 3312 patients received LAAC while 2974 received OAC therapy. On ischemic stroke, no significant difference was observed between OAC and LAAC, according to a random effect model (OR 1.05; 95% CI 0.59-1.84; *p* = 0.88; *I*^2^=61%) (Fig. 8).

#### Hemorrhagic strokes

**Fig. 9:**
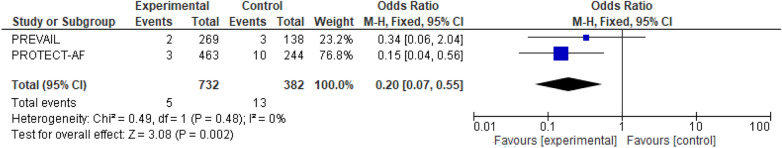
The forest plot of the hemorrhagic strokes

There are two RCT studies mentioned about risk of hemorrhagic strokes, involving 1114 patients were identified, 732 patients received LAAC while 382 received OAC therapy. A fixed effect model's results (Fig 9) showed that LAAC was substantially linked to a decreased risk of hemorrhagic stroke when compared to OAC (OR 0.20, 95% CI 0.07-0.55; p = 0.002; *I*^2^=0%).

#### Systemic embolization

**Fig. 10:**
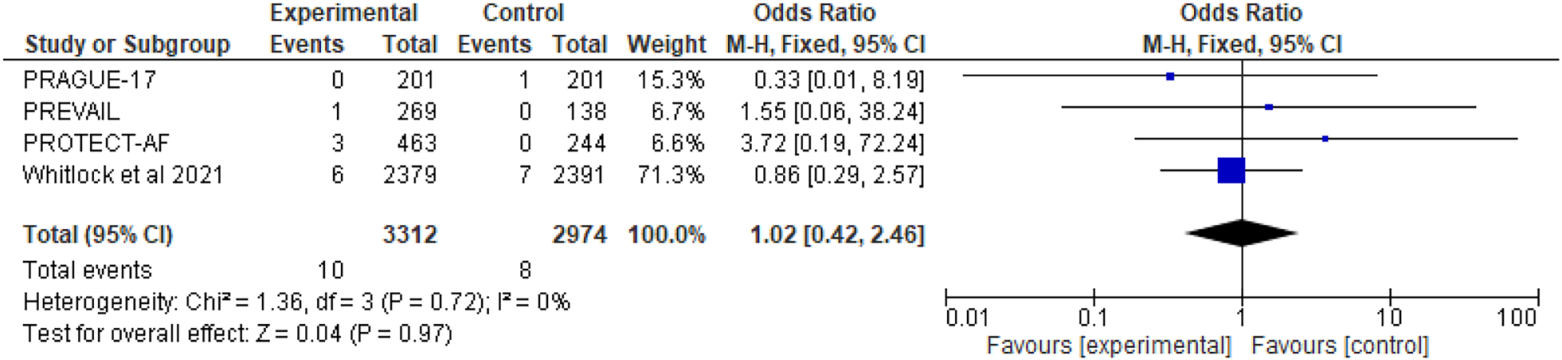
The forest plot of the systemic embolization

There are four RCT studies mentioned about risk of systemic embolization, involving 6286 patients were identified, 3312 patients received LAAC while 2974 received OAC therapy. On systemic embolization, no significant difference were observed between OAC and LAAC, according to a fixed effect model (OR 1.02; 95% CI 0.42-2.46; *p* = 0.97; *I*^2^=0%) (Fig. 10).

## Discussion

AF is the most common arrhythmia worldwide [15]. Its prevalence is estimated to be 7.5 million by 2050 in the USA [16]. Asymptomatic patients with AF can present with stroke as the first symptom of the disease [17]. Hence, OAC is indicated in patients with AF and CHADVASC score > 1 regardless of their symptoms to reduce the risk of stroke. Despite receiving OAC, stroke occurs in up to 5% of patients with AF [11]. When compared to OAC, LAAC was substantially related with a reduced risk of both a hemorrhagic stroke and an all stroke. This meta-analysis's final findings comprised four RCT investigations totaling 6,286 individuals. Between the two groups, there was no discernible difference in ischemic stroke or systemic embolization.

Because anticoagulation works so well, it is the cornerstone of stroke prevention for individuals with atrial fibrillation. For those who cannot tolerate anticoagulation or do not choose to take it long term, LAAC has emerged as a potential alternative. Percutaneous LAAC is advised by the European Society of Cardiology (ESC) guidelines exclusively in situations where anticoagulation is not recommended. Surgical closure of the left atrial appendage lowers the risk of stroke differently from anticoagulants, and the advantages appear to exceed those of oral anticoagulation. To some extent, these cumulative advantages are further enhanced by the technique, which offers continuous and permanent protection against embolic stroke [16–18].

Our aggregated data showed that the LAAC group had a significantly lower risk of hemorrhagic stroke as well as all strokes. The prior meta-analysis by Al-abcha et al. found that the LAAC group consistently had a decreased incidence of hemorrhagic stroke and all strokes [19]. The risk of stroke can be decreased by a variety of variables, and as people age, their risk increases due to an increase in comorbidities. This can be partially explained by the decline in the incidence of hemorrhagic stroke [20]. OAC is usually associated with a major incidence of hemorrhagic stroke that is mostly unraveled by the continuous exposition to the intrinsic bleeding risk carried by OAC. The reason behind this result could be explained by the fact that almost all patients were elderly populations [21]. Between the OAC and LAAC groups, there were similar rates of ischemic stroke and systemic embolization. This is in line with the PRAGUE17 RCT studies that compared efficacy and safety of LAAC device versus DOACs in 402 high-risk patients with AF. At 19.9 months of follow up, the composite of stroke, systematic embolism, major bleeding, cardiovascular death, or procedural complications was similar between the two treatment group [8]. These findings could be connected to regional issues with the LAAC device, such thrombus formation on the device or partial LAA blood flow restriction [22].

This study has significant shortcomings. Initially, all of the study data was used to construct the pooled analysis instead of using specific patient data. One significant drawback was that the studies that were included used different kinds of devices, which made it hard to compare the outcomes according to the kind of device.

## Conclusions

Our findings showed that LAAC was significantly associated with a lower incidence of hemorrhagic stroke and stroke in general when compared to OAC. Our results highlight the need for more research, especially RCT studies or RCT-based systematic reviews and meta-analyses.

## Data Availability

Not applicable.
